# Long Noncoding RNA from PVT1 Exon 9 Is Overexpressed in Prostate Cancer and Induces Malignant Transformation and Castration Resistance in Prostate Epithelial Cells

**DOI:** 10.3390/genes10120964

**Published:** 2019-11-22

**Authors:** Gargi Pal, Jeannette Huaman, Fayola Levine, Akintunde Orunmuyi, E. Oluwabunmi Olapade-Olaopa, Onayemi T. Onagoruwa, Olorunseun O. Ogunwobi

**Affiliations:** 1 Department of Biological Sciences, Hunter College of The City University of New York, New York, NY 10065, USA; 2 Nuclear Medicine Department, College of Medicine, University of Ibadan, Ibadan 200222, Nigeria; 3 Department of Surgery, Urology division, College of Medicine, University of Ibadan, Ibadan 200222, Nigeria; 4 Joan and Sanford I. Weill Department of Medicine, Weill Cornell Medicine, Cornell University, New York, NY 10021, USA

**Keywords:** PVT1 exon 9, prostate cancer, malignant transformation, castration resistance

## Abstract

Prostate cancer (PCa) is the most common non-cutaneous cancer and second leading cause of cancer-related death for men in the United States. The nonprotein coding gene locus plasmacytoma variant translocation 1 (PVT1) is located at 8q24 and is dysregulated in different cancers. PVT1 gives rise to several alternatively spliced transcripts and microRNAs. There are at least twelve exons of PVT1, which make separate transcripts, and likely have different functions. Here, we demonstrate that PVT1 exon 9 is significantly overexpressed in PCa tissues in comparison to normal prostate tissues. Both transient and stable overexpression of PVT1 exon 9 significantly induced greater prostate epithelial cell migration, as well as increased proliferation and corresponding proliferating cell nuclear antigen (PCNA) expression. Notably, implantation into mice of a non-tumorigenic prostate epithelial cell line stably overexpressing PVT1 exon 9 resulted in the formation of malignant tumors. Furthermore, PVT1 exon 9 overexpression significantly induced castration resistance. Consequently, PVT1 exon 9 expression is important for PCa initiation and progression, and holds promise as a therapeutic target in PCa.

## 1. Introduction

Prostate cancer (PCa) is the second leading cause of cancer-related death in men in the United States [1]. According to the American Cancer Society, approximately 174,650 new cases of PCa will be diagnosed in 2019 and will result in 31,620 deaths [2]. On a more global scale, it was reported that PCa had the highest incidence rate for men in 103 countries or territories in 2015 [3].

Long noncoding RNAs (lncRNAs) play an important role in various biological and pathological processes frequently implicated in cancer, including proliferation, apoptosis, cell cycle progression, migration, and invasion [4]. Plasmocytoma variant translocation 1 (PVT1) is a long nonprotein-coding gene located at the 8q24 human chromosomal region and is commonly dysregulated in PCa [5,6]. The PVT1 gene locus is comprised of at least twelve exons, which gives rise to several alternatively spliced nonprotein coding transcripts, and encodes six microRNAs: miR-1204, miR-1205, miR-1207-3p, miR-1207-5p, and miR-1208 [7]. PVT1 has been found to be overexpressed in various cancers including breast, lung, colorectal, ovarian, and PCa [8,9,10,11,12]. Numerous studies have confirmed that PVT1 upregulation is associated with tumor progression and reduced survival in several types of cancer [13,14]. Located 50 kb upstream of PVT1 is the MYC gene [15]. Studies have shown that PVT1 and MYC are often coamplified in several solid tumors [15]. Coamplification and cooperation between c-MYC and PVT1 genes have been found in malignant pleural mesothelioma [16], ovarian and breast cancer [17], as well as neuroblastoma [18].

Recent studies have identified PVT1 as being procarcinogenic and have shown that PVT1 amplification increases the risk of PCa incidence [5,9,19]. We previously demonstrated that the transcript from exon 9 of PVT1 may be involved in PCa, but the underlying molecular mechanisms were unknown [7].

In this study, we sought to uncover the role of PVT1 exon 9 in PCa. We assessed its expression in histologically confirmed normal prostate and PCa tissues. Our results confirmed significant overexpression of PVT1 exon 9 in PCa tissues in comparison to normal prostate tissue. Furthermore, both transient and stable overexpression of PVT1 exon 9 in a nontumorigenic prostate epithelial cell line induced increased cell proliferation and migration, which are characteristics of the tumor phenotype. Notably, a stable subline of the nontumorigenic prostate epithelial cell line overexpressing PVT1 exon 9 demonstrates increased cell proliferation, and migration, as well as in vivo malignant tumor growth and castration resistance. Interestingly, histopathological analysis showed that the malignant neoplasm formed has features of aggressive and invasive PCa. This is the first direct demonstration of an alternatively spliced transcript from the PVT1 gene locus possessing oncogenic capability. The data presented here show that PVT1 exon 9 has a role in cancer initiation, progression, and castration resistance in PCa, and may have clinical applications as a therapeutic target or a diagnostic biomarker.

## 2. Materials and Methods

### 2.1. Tissue Samples

Human Subjects Research: The City University of New York Institutional Review Board approved the study (approval number 2017-1144). The animal study was approved under protocol number 2015-0038 on 10 June 2019, while the human tissue study was approved on 8 August 2016 under protocol number 2016-0368. Informed consent was obtained whenever required by the IRB-approved protocol.

Normal prostate tissue (*n* = 22) and PCa tissue (*n* = 28) samples were obtained following informed consent from patients who had undergone prostatectomy or transrectal ultrasound-guided biopsy at the University College Hospital, Ibadan, Nigeria. Tissues were collected in compliance with the University of Ibadan-University College Hospital Ethics Committee and City University of New York Institutional Review Board approved protocols, and histopathological analysis was performed.

### 2.2. Cell Culture

The nontumorigenic prostate epithelial cell line, RWPE-1, was cultured in keratinocyte serum free medium (SFM) supplemented with 0.05 mg/mL bovine pituitary extract (BPE), 5 ng/mL epidermal growth factor (EGF), and 1% penicillin/streptomycin (P/S). The castration-resistant PCa cell line, 22RV1, was grown in RPMI-1640 supplemented with 10% heat inactivated FBS, and 1% P/S. The castration-resistant PCa cell line, C4-2B, was cultured in DMEM supplemented with 200 mL Ham’s F12, 10% heat-inactivated FBS, 1% penicillin/streptomycin, insulin (5 µg/mL), triiodothyronine (13.65 pg/mL), human apo-transferrin (4.4 µg/mL), d-Biotin (0.244 µg/mL), and Adenin (12.5 µg/mL).

### 2.3. Transfections

RWPE1 cells were seeded in six-well plates. To investigate the role of PVT1 exon 9, the transcript from PVT1 exon 9 was cloned into the mammalian expression vector pcDNA3.1 (Invitrogen, Carlsbad, CA, USA). After reaching 60–70% confluence, media was replaced with Opti-MEM (Thermo Fisher Scientific Inc.; Wilmington, DE, USA) and cells are transfected with 100 ng of plasmid construct using Lipofectamine 3000 (Thermo Fisher Scientific Inc.; Wilmington, DE, USA), according to the manufacturer’s instructions. Transfected cells were then incubated at 37 °C for 24 h, after which the media was replaced with cell line—specific culture media. For knock down experiments, transfections were done using PVT1 exon 9 small interfering RNAs (siRNAs) (Sigma, St, Louis, MO, USA) at 30 pM final concentration per well using lipofectamine RNAiMax (Invitrogen Inc., Carlsbad, CA, USA) in Opti-MEM (1×) reduced serum media (Gibco, Gaithersburg, MD, USA). A nonspecific (scramble) control siRNA was also transfected at the same concentration as the negative control into control cells. Cells were incubated at 37 °C for 72 h. The sequence of siRNAs used are indicated in Table 1.

### 2.4. Cloning of PVT1 Exon 9 to Make Stable Cell Line

The PVT1 exon 9 fragment was synthesized by IDT (USA) and the dsDNA was reconstituted and amplified with polymerase chain reaction (PCR). The forward primer contained HindIII, and reverse primer contained BamHI restriction site. The PCR was performed as follows: initial denaturation of DNA at 98 °C for 30 s, an amplification program consisting of 30 cycles at 98 °C for 10 s, 55 °C for 30 s, and 72 °C for 30 s and the final extension of 1 cycle at 72 °C for 10 min. PCR product was resolved by gel electrophoresis on a 1.8% agarose gel in Tris–acetate buffer (1×). The PCR product was purified with a gel purification kit (Qiagen, Hilbert, Germany) and digested with HindIII and BamHI restriction endonucleases. The resulting gene fragment was purified and ligated into pcDNA3.1(+) vector (Addgene, Cambridge, MA, USA) digested with the same enzymes. The ligation mixture was transformed into *E. coli* JM109 competent cells as described by Sambrook et al. [20] and the recombinant plasmid was confirmed by restriction digestion by HindIII and BamHI, colony PCR as well as by sequencing. For stable cell line selection, prostate epithelial cell line (RWPE1) transfected with PVT1 exon 9 or empty pcDNA3.1 vector was grown in the presence of geneticin (Gibco, Gaithersburg, MD, USA) at a concentration of 100 μg/mL for two weeks.

### 2.5. RNA Extractions

At 75% confluency, total RNA was extracted from nontransfected and transfected RWPE1 cells grown in 75 cm^2^ flasks using RNeasy Mini Kit (Qiagen, Germany, cat# 74104). After quantification with a Nanodrop1000 spectrophotometer (NanoDrop, Madison, WI, USA), 1 μg of RNA was reverse-transcribed into complementary DNA (cDNA) using QuantiTect reverse transcription kit (Qiagen, Germany, cat# 205311). The reverse transcription primer mix contains a specially optimized mix of oligo-dT and random primers that enable cDNA synthesis from all regions of RNA transcripts.

### 2.6. Quantitative Reverse Transcriptase Polymerase Chain Reaction (qPCR)

The qPCR assays were performed on an ABI 7500 platform (Applied Biosystems instruments, Grand Island, NY, USA)) with 25 μL reaction volumes containing 12.5 μL SYBR Green PCR master mix (Life Technologies, Grand Island, NY, USA cat# 4309155), 0.4 µM final concentration for primers (Forward Primer: 5′ CATGACTCCACCTGGACCTT 3′ and Reverse primer: 5′ GTGGGCGATGAAGTTCGTA 3′), 2.5 μL cDNA template, and 7.5 μL of water. The thermal cycle protocol used was as follows: 50 °C for 2 min, 10 min initial denaturation at 95 °C, and 40 cycles of 15 s denaturation at 94 °C, 1 min annealing at 58 °C. GAPDH was used as housekeeping gene for all the qPCR experiments. Relative gene expression was calculated using the comparative CT method known as 2^ΔΔCt^.

### 2.7. Migration Assays

Wound healing migration assays were performed as previously described [21]. A total of 10^5^ cells were seeded into six-well plates. At 80% confluency, the cell monolayer was wounded with a 200 μL pipette, washed with PBS and medium replaced. Images were taken at 0 h, 24 h, 48 h, and 72 h intervals. Images were taken using Motic Images Plus v.2.0 Software (Motic, Richmond, BC, Canada).

### 2.8. Cell Proliferation Assays

A total of 10^4^ cells were seeded into 96 well plates. At 60–70% confluency, the cells were transfected with PVT1 exon 9. After 24 h, MTT cell proliferation assays were performed and absorbance measured at 490 nm with a microplate reader (Spectramax i3 multimode microplate reader, USA).

### 2.9. Western Blotting

Total protein was extracted using a cocktail of RIPA lysis buffer (VWR Life Science, cat# N653-100ML), protease inhibitor tablets (Thermo scientific, cat# A32953), and Phenyl methylsulfonyl fluoride (Amresco, cat# M145-5G). The protein concentration was determined with Bradford reagent (Biorad, cat# 500-0205). Primary antibodies used were against PCNA (2586S, Cell signaling Technology, Danvers, MA, USA) and α tubulin (SC32293, Santa Cruz Biotechnology, Dallas, TX, USA). Secondary antibodies used were either against mouse or rabbit (Sigma-Aldrich, St. Louis, MO, USA), as appropriate. Li-COR Odyssey CLx with infrared fluorescence, IRDye secondary antibodies and imagers were used to detect western blots without film or chemiluminescent substrates. All antibodies were made in 5% BSA. The primary antibodies were prepared using 1:1000 dilution and incubated overnight at 4 °C. For secondary antibodies, we used 1:10,000 dilution and incubated for 2 h at room temperature. Western blots were analyzed and quantified with Odyssey imager; Image Studio version 5. α tubulin was used as loading control.

### 2.10. Xenograft Tumor Studies

Animal Research: Animal studies were approved by Weill Cornell Medicine Institutional Animal Care and Use Committee (IACUC) (approval number 2015-0038). Whenever necessary, euthanasia was performed using carbon dioxide displacement, as approved.

Animal studies were performed using 7 to 8-week-old male NOD-scid IL2Rgnull-3/GM/SF (NSG) mice maintained under specific pathogen-free conditions and used in accordance with protocols (Protocol Number: 2015-0038) approved by the Institutional Animal Care and Use Committee (IACUC) at Weill Cornell Medicine [22]. RWPE1_ex9, RWPE1_ev, and RWPE1 cells were harvested and resuspended in PBS. A total of 3 × 10^6^ cells resuspended in 0.1 mL of PBS was subcutaneously implanted into the flank of each mouse. Five mice each were implanted with either RWPE1_ex9 or RWPE1_ev or RWPE1 cells. Tumor growth was monitored, and tumor sizes were measured every two or three days. Tumor volume was calculated using the formula, volume = (length × width^2^ × 0.5). Mice were euthanized with CO_2_ displacement. All organs were fixed in 10% neutral buffered formalin, and decalcification of select organs was performed in formic acid solution (Surgipath Decalcifier I, Leica Biosystems, Wetzlar, Germany). Tissues were processed and embedded in paraffin in a Leica ASP6025 tissue processor. Blocks were sectioned at 5 microns, stained with hematoxylin and eosin (H&E), and examined by a board-certified veterinary pathologist. All animal experimentations were approved by the Laboratory of Comparative Pathology, Center of Comparative Medicine and Pathology at Memorial Sloan Kettering Cancer Center.

### 2.11. Assessment of Castration Resistance

RWPE1, RWPE1_ev, RWPE1_ex9 cells were seeded in 96-well plates at a density of 10^4^ cells/well and then treated with 10 μM, 25 μM, and 50 μM of abiraterone acetate. To check responsiveness or resistance to abiraterone acetate, cell viability was assessed after 24 h using MTT assays. Absorbance at 490 nm was measured with a microplate reader (Spectramax i3 multimode microplate reader, USA).

### 2.12. Statistical Analysis

Data was always collected from at least three independent experiments. All results are presented as mean ± standard error of the mean (SEM). Analysis of statistical significance of differences between groups was performed using two-tailed Student’s *t*-test, and only values with *p* < 0.05 were deemed significant. For comparison of variables, a Student’s *t* test or analysis of variance (ANOVA) test were used for analysis of each set of continuous and categorical data. Analysis of variance (ANOVA) was performed using the SPSS Statistics software (http://www-01.ibm.com/software/analytics/spss/) on normalized data. *p* values < 0.05 were considered significant.

## 3. Results

### 3.1. Expression of PVT1 Exon 9 Is Upregulated in PCa Tissues

**Figure 1:**
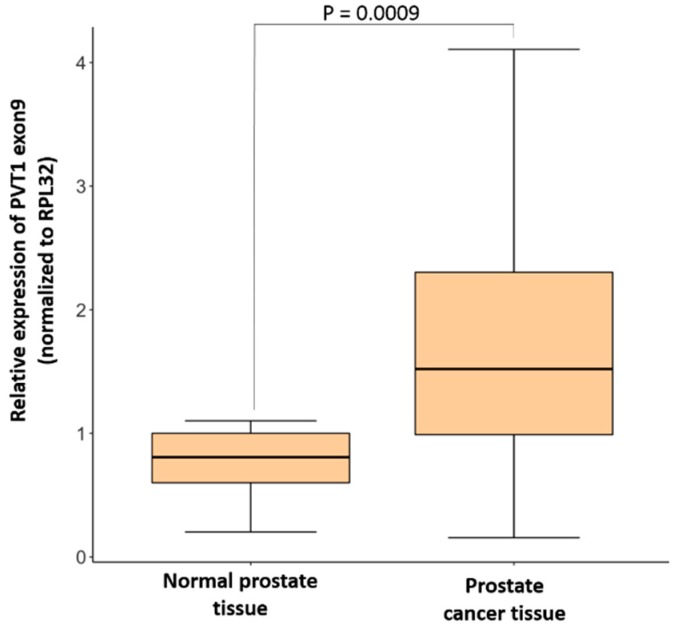
PVT1 exon 9 expression is significantly higher in prostate cancer tissue (*n* = 28) in comparison to normal prostate tissues (*n* = 22). Data are presented as mean +/− standard error of the mean (SEM). Statistical differences were determined with one-way ANOVA. All the criteria for significance were set at *p* < 0.05.

To determine PVT1 exon 9 expression in clinical specimens, PVT1 exon 9 was analyzed in histologically confirmed normal prostate (*n* = 22) and PCa tissue (*n* = 28). Samples were obtained from males who had undergone a prostatectomy or a transrectal ultrasound-guided biopsy. RNA extraction, cDNA synthesis and real-time quantitative polymerase chain reaction (qPCR) were performed to assess PVT1 exon 9 expression in these tissues. We observed that mean expression of PVT1 exon 9 in PCa tissues is significantly higher than in normal prostate tissue, as seen in Figure 1.

### 3.2. PVT1 Exon 9 Promotes Increased Cell Proliferation and Migration

**Figure 2:**
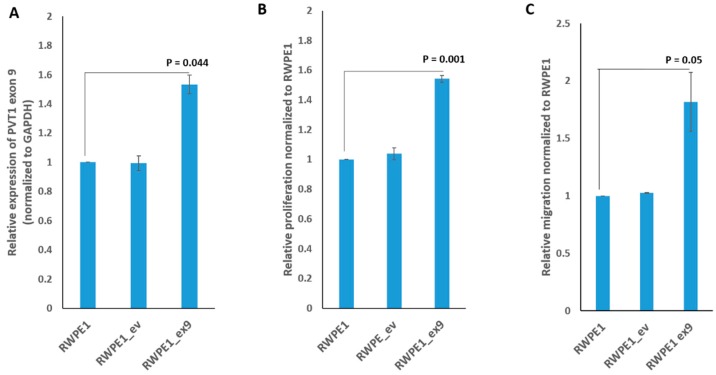
PVT1 exon 9 stable cell line induces gene expression, prostate epithelial cell proliferation, and migration. (**A**) PVT1 exon 9 expression, (**B**) cell proliferation, (**C**) cell migration. Data are presented as mean +/− standard error of the mean (SEM). All the criteria for significance were set at *p* < 0.05. All experiments were done three different times. qPCR was performed in quadruplicates, using RNA from cells from three different passages.

A plasmid containing PVT1 exon 9 and the empty plasmid vector were separately transfected into RWPE1, a nontumorigenic prostate epithelial cell line. Effects of transient and stable overexpression of PVT1 exon 9 on cell proliferation and migration were assessed. As shown in Supplementary Figure S1, cells transiently overexpressing PVT1 exon 9 showed significantly increased proliferative and migratory capacity in comparison to cells containing the empty vector, and untransfected RWPE1 cells. Furthermore, we successfully made a stable subline overexpressing PVT1 exon 9 (RWPE1_ex9) and confirmed stable overexpression of PVT1 exon 9 in RWPE1_ex9, as seen in Figure 2A. Notably, RWPE1_ex9 is significantly more proliferative and more migratory than RWPE1 with empty vector (RWPE1_ev), and untransfected RWPE1 cells, as seen in Figure 2B,C.

### 3.3. PVT1 Exon 9 Regulates Proliferating Cell Nuclear Antigen (PCNA) Expression

**Figure 3:**
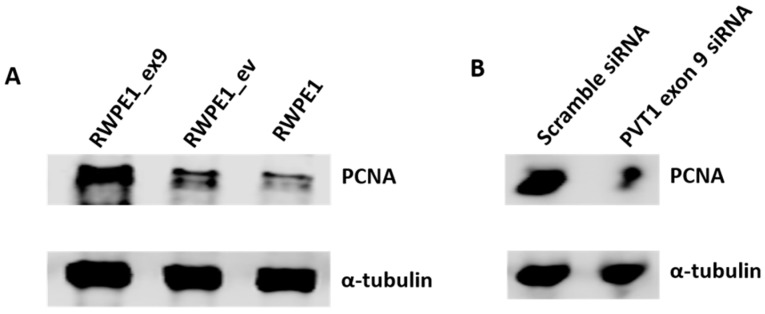
PVT1 exon 9 regulates the expression of proliferating cell nuclear antigen (PCNA). (**A**) Expression of PCNA is upregulated with PVT1 exon 9 overexpression and (**B**) downregulated with PVT1 exon 9 silencing. Western blotting experiments were performed two separate times, image presented is representative.

We hypothesized that PVT1 exon 9 may be exerting its effects on cell proliferation through regulation of proliferating cell nuclear antigen (PCNA) expression. To determine this, we performed Western blotting using the cell lysates of RWPE1, RWPE1_ev and RWPE1_ex9. We found that PCNA is overexpressed in RWPE1_ex9 compared to RWPE1 and RWPE1_ev. Additionally, specific silencing of PVT1 exon 9 in the MDA PCa 2b prostate cancer cell line using a PVT1 exon 9 specific siRNA resulted in decreased PCNA expression. Thus, confirming that PVT1 exon 9 regulates PCNA expression, as seen in Figure 3A,B.

### 3.4. PVT1 Exon 9 Initiates Carcinogenesis In Vivo

**Figure 4:**
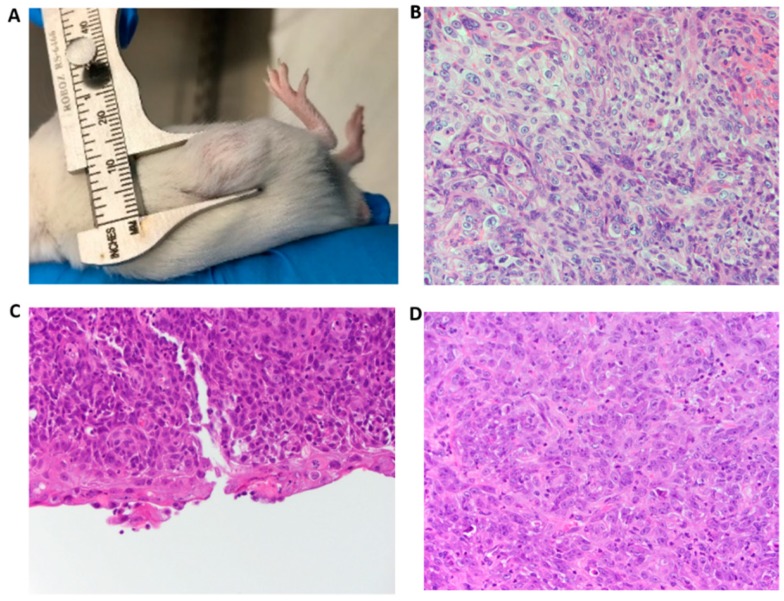
PVT1 exon 9 induces in vivo cancer initiation. (**A**) Tumor in mouse after implantation of RWPE1_ex9. (**B**–**D**) Histological analysis (H&E staining) revealed that the tumor exhibited whorled appearance containing large pleomorphic, and hyperchromatic nuclei in mouse 1, 2, and 3, respectively (40× objective).

To determine if induction of the cancer phenotype in vitro by PVT1 exon 9 is recapitulated in vivo, we performed experiments with age-matched male NOD SCID γ (NSG) mice. To this end, RWPE1_ex9, RWPE1_ev, and RWPE1 cells were subcutaneously implanted into the flank of age-matched NSG mice. Interestingly, male NSG mice implanted with PVT1_ex9 progressively developed visible tumors. Whereas, no tumors developed in age-matched male NSG mice implanted with same number of RWPE1 or RWPE1_ev cells. These data clearly demonstrate the tumor initiating capability of PVT1 exon 9, as seen in Figure 4A–D; Supplementary Figures S2–S4. Histopathological analysis of the tumors formed from RWPE1_ex9 cells confirmed that the tumors were a densely cellular, unencapsulated mass composed of neoplastic epithelial cells. These cells were arranged in islands, supported by a moderately dense fibrovascular stroma, and the tumor had infiltrated the surrounding subcutaneous tissue. The neoplastic cells had a high degree of anaplasia, with marked anisocytosis. Thus, these were malignant tumors. The expansible but also invasive nature of the tumor could be seen in tumor invading adjacent skeletal muscle, as seen in Supplementary Figure S2B. There was, however, an absence of prostatic glandular differentiation. Further, they showed squamous differentiation with early keratinization and the presence of keratohyalin granules. The mitotic rate exceeded 5 mitoses per 400× high-powered field.

### 3.5. PVT1 Exon 9 Induces Castration Resistance

**Figure 5:**
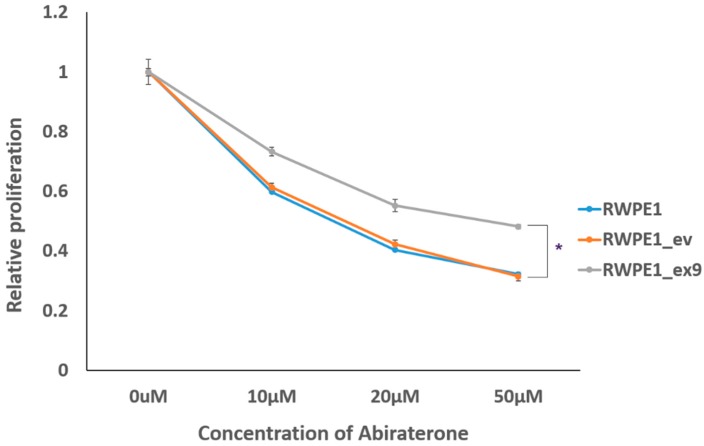
PVT1 exon 9 confers castration resistance. RWPE1_ex9 showed increased abiraterone resistance in comparison to RWPE1 or RWPE1_ev. Data are presented as mean +/− standard error of the mean (SEM). Statistical differences were determined with one-way ANOVA. All the criteria for significance were set at *p* < 0.05. Experiments were done three different times.

Finally, we investigated whether induction of malignant transformation by PVT1 exon 9 had also conferred castration resistance onto RWPE1_ex9. To do this, we examined the effect of increasing concentrations of abiraterone (a second generation androgen deprivation therapy in clinical use) on the viability of RWPE1, RWPE1_ev, and RWPE1_ex9 cells. Increasing concentrations of abiraterone progressively inhibited the viability of RWPE1 and RWPE1_ev cells. However, RWPE1_ex9 cells retained significantly more cell viability than RWPE1_ev and RWPE1 cells. Thus, indicating that PVT1 exon 9 induces the acquisition of castration resistance, as seen in Figure 5. Moreover, PVT1_ex9 demonstrates a similar behavioral pattern to C4-2B and 22RV1, well-established metastatic castration-resistant PCa cell lines, as seen in Supplementary Figure S5.

## 4. Discussion

Located at the 8q24 chromosomal region, the long nonprotein-coding gene PVT1 has been shown to be overexpressed in various cancers including PCa, breast, pancreatic, lung, and gastric cancers. Downregulation of expression of the long noncoding RNA (lncRNA) from PVT1 inhibits proliferation and migration by regulating p38 expression in PCa [19]. Increasing evidence has shown the important role of lncRNAs in many different biological processes [23,24,25,26,27,28,29,30,31]. PVT1 itself has been implicated in cell proliferation, apoptosis, lymph node invasion, metastasis, and tumor prognosis [23]. Importantly, PVT1 has been associated with poorer prognosis and regulation of tumor growth in PCa [31]. PVT1 is a very long noncoding RNA with many alternatively spliced transcripts from at least 12 exons. No previous study has investigated the function of any alternatively spliced transcript of PVT1.

For the first time, we are reporting that the transcript from PVT1 exon 9 is significantly overexpressed in PCa tissues in comparison to normal human prostate tissues. Our data provide the first description of the function of a single alternatively spliced transcript from the PVT1 gene locus. Notably, we provide direct proof that the long noncoding RNA from PVT1 exon 9 has oncogenic activity.

Here, we report that transient overexpression of PVT1 exon 9 induces cell proliferation and migration. Notably, we have established a subline (RWPE1_ex9) of a nontumorigenic prostate epithelial cell line (RWPE1) now stably overexpressing PVT1 exon 9. Not surprisingly, stable overexpression of PVT1 exon 9 in RWPE1_ex9 similarly results in increased proliferative and migratory capability by prostate epithelial cells. This enhanced proliferative capability conferred by PVT1 exon 9 is likely due to regulation of expression of PCNA. Accordingly, prostate epithelial cells stably overexpressing PVT1 exon 9 (RWPE1_ex9) have increased expression of PCNA in comparison to wild type cells or cells with the empty vector. Silencing of PVT1 exon 9 expression in a well-established PCa cell using specific siRNAs produced the exact opposite effect: inhibition of PCNA expression. Thus, confirming that PVT1 exon 9 regulates PCNA expression. It is possible that PVT1 exon 9 regulation of PCNA is a mechanism by which PVT1 exon 9 regulates the cell cycle, and consequently cell proliferation. Further, siRNA studies revealed that knocking down expression of PVT1 exon 9 significantly decreases the expression of other PVT1 exons. Whereas knocking down the expression of other PVT1 exons did not significantly affect expression of PVT1 exon 9. This indicates that PVT1 exon 9 is a major regulatory part of the PVT1 gene, as seen in Supplementary Figure S6, and this may explain the critical role of PVT1 exon 9 in inducing malignant transformation and carcinogenesis of prostate epithelial cells.

It is most noteworthy that PVT1 exon 9 is capable of PCa initiation. In the current study, we have confirmed that PVT1 exon 9 has a tumorigenic and malignant transformative effect in vivo on prostate epithelial cells. Notably, the morphologic appearance of the malignant neoplasms by PVT1 exon 9 overexpressing prostate epithelial cells was akin to prostatic carcinoma with squamous metaplasia. The not only expansible, but also invasive nature of the tumor could be seen in tumor invading adjacent skeletal muscle. Importantly, histopathological analysis confirmed that the neoplastic cells demonstrated a high degree of anaplasia, thus confirming the cancerous nature of the tumors. These data give evidence that PVT1 exon 9 has an important role in PCa initiation and invasion.

Androgen receptor (AR) signaling is widely considered to be the most important molecular signaling pathway for PCa. And androgen deprivation therapy (ADT) is the most common therapeutic strategy offered to patients with locally advanced and metastatic PCa [32]. While a majority of patients initially respond to ADT, most will eventually develop castration resistance [33]. When PCa patients relapse after ADT, the disease is said to have progressed to castration-resistant prostate cancer (CRPC). In CRPC, second generation ADTs like abiraterone acetate are commonly used and they are generally effective [34]. Abiraterone acetate inhibits androgen biosynthesis and has been widely used to treat hormone-sensitive metastatic PCa [35]. However, some CRPC patients are initially unresponsive to second generation ADTs, such as abiraterone acetate. Other CRPC patients may develop secondary resistance to abiraterone acetate after initial responsiveness. This is a very important clinical problem for which an urgent solution is required. The solution to either primary or secondary castration resistance in CRPC patients on secondary generation ADTs like abiraterone lies in understanding the molecular mechanisms of resistance to abiraterone. Here, we make a significant contribution to addressing this important clinical problem by demonstrating, for the first time, that PVT1 exon 9 overexpression confers resistance to abiraterone acetate. Thus, indicating that PVT1 exon 9 overexpression is a novel mechanism of castration resistance, and hence progression, in PCa.

In conclusion, our results indicate that PVT1 exon 9 is overexpressed in PCa. This study has provided novel evidence that PVT1 exon 9 is able to induce increased cell proliferation and migration of prostate epithelial cells. The observed PVT1 exon 9-induced hyperproliferation may be accomplished through its observed regulatory effect on PCNA expression. Importantly, PVT1 exon 9 overexpression is able to induce malignant transformation of prostate epithelial cells and PCa initiation in vivo. The observed PVT1 exon 9-dependent tumors were invasive and expansile prostatic carcinomas with squamous metaplasia. Of significant clinical relevance, overexpression of PVT1 exon 9 in prostate epithelial cells confers castration resistance. Consequently, we report for the first time, that an alternatively spliced long noncoding transcript from PVT1 exon 9 is involved in PCa initiation, invasion, and castration resistance. Therefore, PVT1 exon 9 may have clinical applications as a diagnostic and prognostic biomarker, or as a therapeutic target in PCa.

## Figures and Tables

**Table 1:** Sequence of PVT1 siRNAs.

Name	Sequence (5′ to 3′)
Scamble siRNA Forward	CUCACUACCGUCGACCCCAUU
Scamble siRNA Reverse	UGGGGUCGACGGUAGUGAGUU
PVT1 exon 9 siRNA Forward	ACCUAUGAGCUUUGAAUAA
PVT1 exon 9 siRNA Reverse	UUAUUCAAAGCUCAUAGGU

## Supplementary Materials

The following are available online at https://www.mdpi.com/2073-4425/10/12/964/s1, Supplementary Figure S1: PVT1 exon 9 promotes prostate epithelial cell proliferation and migration, Supplementary Figure S2: Tumor growth in mouse 1, Supplementary Figure S3: Tumor growth in mouse 2, Supplementary Figure S4: Tumor growth in mouse 3, Supplementary Figure S5: PVT1 exon 9 confers castration resistance, Supplementary Figure S6: PVT1 exon 9 regulates expression of other PVT1 exons.
