# Supplementary material for: Long Noncoding RNA from PVT1 Exon 9 Is Overexpressed in Prostate Cancer and Induces Malignant Transformation and Castration Resistance in Prostate Epithelial Cells
Source: Genes (Basel). 2019 Nov 22;10(12):964. doi: 10.3390/genes10120964 (PMC6969942; doi:10.3390/genes10120964)
Supplement: Supplementary file 1 [file genes-10-00964-s001.pdf]

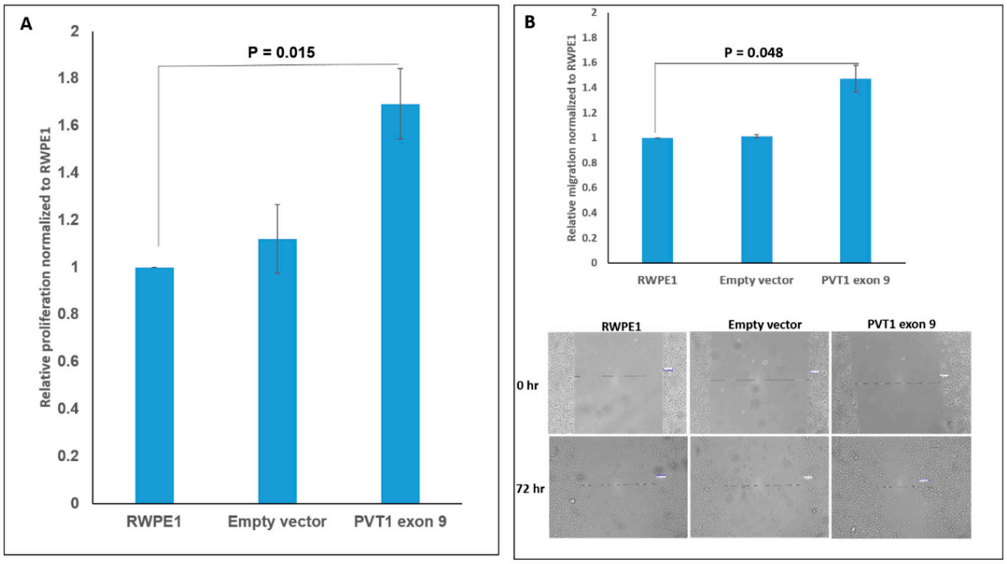

Supplementary Figure S1. PVT1 exon 9 promotes prostate epithelial cell proliferation and migration. (A) cell proliferation, (B) migration. Data is presented as mean +/− standard error of the mean (SEM). All the criteria for significance were set at $P < 0.05$.

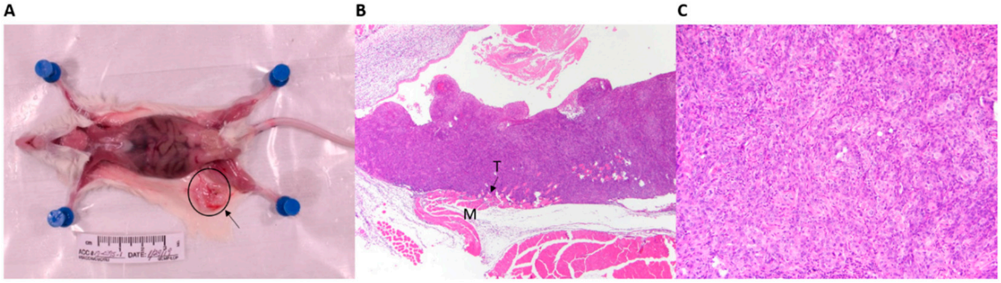

Supplementary Figure S2. Tumor growth in mouse 1. (A) Tumor in mouse 1 after implantation of RWPE1_ex9. (B) Histopathological analysis showing that tumor cells are expansible and invading adjacent muscles (X4 objective). (C) H&E staining revealed that tumor cells are composed of large, pleomorphic and hyperchromatic nuclei (X20 objective).

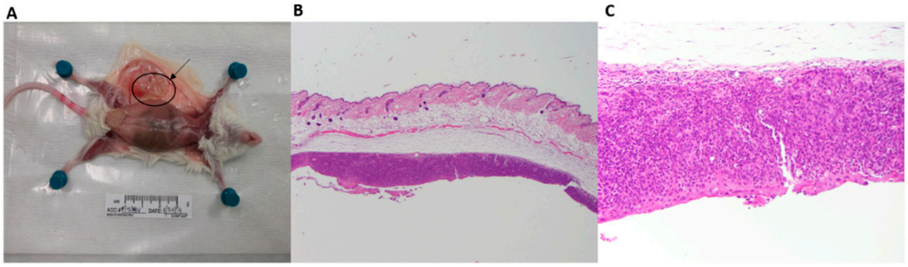

Supplementary Figure S3. Tumor growth in mouse 2. (A) Tumor in mouse 2 after implantation of RWPE1_ex9. (B) Histopathological analysis showing typical characteristic features of anisonucleosis with moderate amount of cytoplasm (X4 objective). (C) H&E staining revealed that tumor cells are composed of large, pleomorphic and hyperchromatic nuclei (X20 objective).

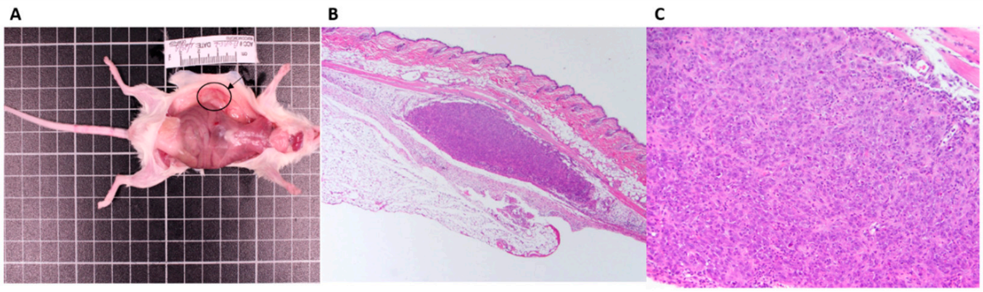

Supplementary Figure S4. Tumor growth in mouse 3. (A) Tumor in mouse 3 after implantation of RWPE1_ex9. (B) Histopathological analysis showing typical characteristic features of anisonucleosis with moderate amount of cytoplasm (X4 objective). (C) H&E staining revealed that tumor cells are composed of large, pleomorphic and hyperchromatic nuclei (X20 objective).

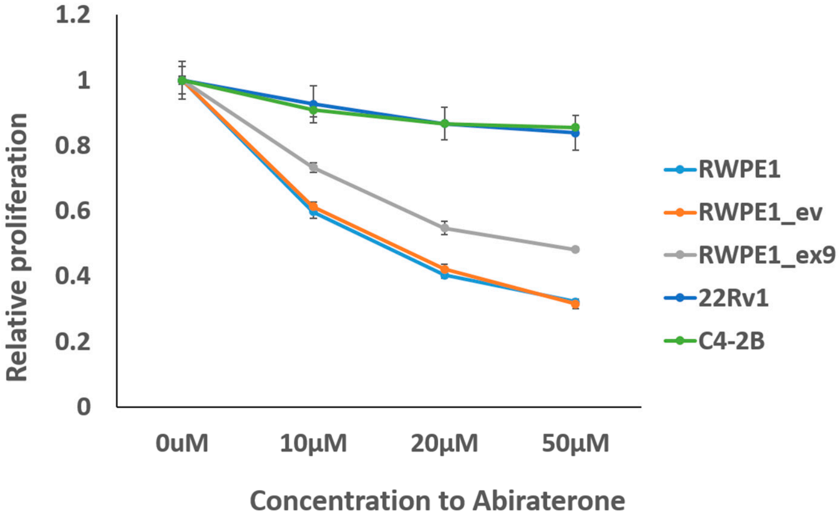

Supplementary Figure S5. PVT1 exon 9 confers castration resistance. RWPE1_ex9 demonstrates significantly increased abiraterone resistance in comparison to RWPE1 or RWPE1_ev, and shows a trend towards the extent of resistance by 22RV1 and C4-2B. Data is presented as mean +/− standard error of the mean (SEM). Experiments were done three different times and statistical differences were determined with one-way ANOVA. All the criteria for significance were set at $P < 0.05$.

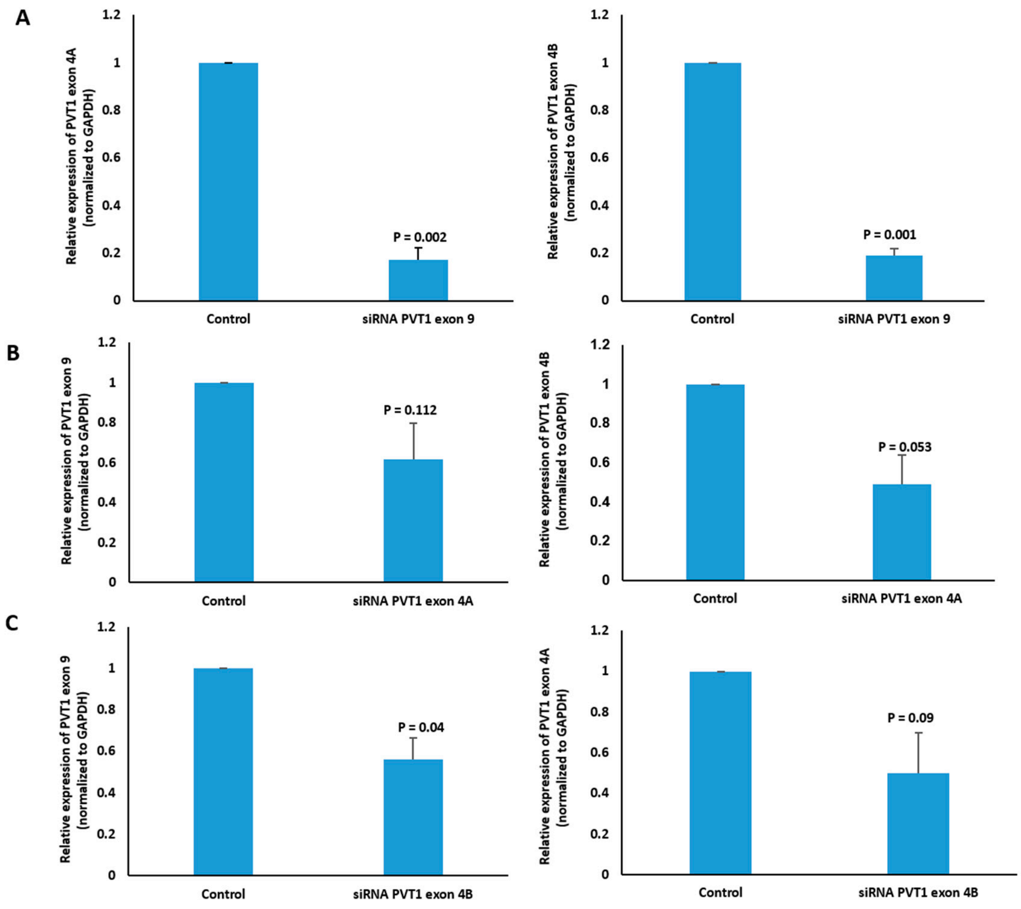

Supplementary Figure S6. PVT1 exon 9 regulates expression of other PVT1 exons. Data is presented as mean ± standard error of the mean. Statistical analysis was performed using the student's t-test. *P* values less than 0.05 were considered significant. GAPDH, glyceraldehyde 3-phosphate dehydrogenase; siRNA, small interfering RNA.
